# Vagus Nerve Stimulation (VNS) in Super Refractory New Onset Refractory Status Epilepticus (NORSE)

**DOI:** 10.1155/2019/7852017

**Published:** 2019-01-21

**Authors:** Mohankumar Kurukumbi, James Leiphart, Anam Asif, Jing Wang

**Affiliations:** ^1^Department of Neurology, Inova Fairfax Hospital, Falls Church, VA, USA; ^2^VCU School of Medicine, Inova Campus, Falls Church, VA, USA; ^3^Department of Neurocritical Care, Inova Fairfax Hospital, Falls Church, VA, USA

## Abstract

The treatment protocol of status epilepticus has many associated toxicities so there is interest in alternate nonmedicinal therapies for managing New Onset Refractory Status Epilepticus (NORSE) patients. Vagus nerve stimulation (VNS) is an FDA-approved therapy for refractory epilepsy that has been shown to decrease the frequency and severity of seizures. We present the case of a patient with new-onset refractory status epilepticus (NORSE) whose seizures were successfully treated with vagus nerve stimulation. A 25-year-old male with no history of epilepsy or other neurological disorders presented with altered mental status and generalized tonic-clonic seizures following a two-week history of an upper respiratory tract infection. Lumbar puncture showed neutrophilic pleocytosis, and he was treated for bacterial and viral meningoencephalitis. In spite of treatment, his seizures began increasing in frequency. On day three, the patient entered status epilepticus (SE) refractory to intensive pharmacotherapy with maximal doses of valproate, levetiracetam, and propofol. On day four, SE remained refractory, so pentobarbital was introduced with targeted burst suppression pattern on electroencephalography (EEG). Patient continued to be refractory to these measures, so a vagus nerve stimulator (VNS) was implanted (day eight). Following VNS implantation, EEG demonstrated significant reduction of seizure activity and subsequent magnet swiping continued aborting electrographic seizures. No SE or electrographic seizures were reported for seventy-two hours, but few occasional discharges were reported. Seizures eventually recurred on day fourteen and the patient succumbed to his multiple comorbidities on day seventeen. Due to the efficacy of VNS in refractory epilepsy, there was interest in using it in refractory status epilepticus. Multiple case reports have described a benefit from implantation of VNS in the treatment of SE. The successful use of VNS to acutely terminate status epilepticus for seventy-two hours in this critically ill patient adds to current evidence that there is utility in using VNS for refractory status epilepticus.

## 1. Introduction

New-onset refractory status epilepticus (NORSE) is a syndrome where status epilepticus manifests in patients who were previously healthy and lack a history of epilepsy or neurological disorders. The treatment protocol is intensive and includes benzodiazepines, anticonvulsants, and eventually anesthetics to medically induce coma when polypharmacy is exhausted [1]. If seizures continue or recur after 24 hours following treatment with anesthetics, it is termed superrefractory status epilepticus (SRSE). Because of high mortality with the polypharmacy and continuous anesthetic use, there has been great interest to use nonmedicinal devices like VNS in aborting a seizure. Here we present a case of super refractory NORSE treated with VNS.

## 2. Case Report

The patient was a 25-year-old male presenting with altered mental status and generalized tonic clonic seizures following a 2-week history of an upper respiratory tract infection. MRI of brain was negative for any acute pathology and nonlesional for seizures.* Results of a paraneoplastic antibody panel were negative but he was found to have neutrophilic pleocytosis on lumbar puncture. Cerebrospinal fluid cultures were negative but he was empirically treated for bacterial and viral meningoencephalitis with ceftriaxone, vancomycin, and acyclovir*. Seizures began increasing in frequency despite frequent treatment with benzodiazepines and, on day three, the patient worsened to have status epilepticus. Treatment with maximal doses of valproate, levetiracetam, and propofol was started but, by day four, the patient's seizures remained refractory. As a result, the patient was placed under a pentobarbital-induced coma with burst suppression pattern on electroencephalography (EEG). On day 8, five days after status and due to continued seizure activity as evidenced by EEG, a VNS was implanted. VNS was turned on with the settings: Output output current 1.5 milliamperes, Pulse Width 500 microseconds, Frequency 30, On time 30 seconds, and Time interval 3 minutes.

**Figure 1:**
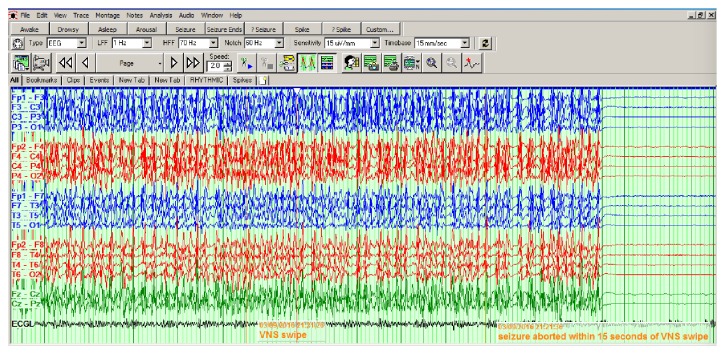
EEG demonstrated cessation of seizure activity within 15 seconds of magnet swiping.

Magnet Output current 2 milliamperes, On time 60 seconds.* Three days after implantation of VNS, there was generalized suppression of EEG activity with continued use of pentobarbital; however, the patient continued to have electrographic seizures which were successfully aborted by the VNS magnet swiping (see Figure 1)*. No other changes were made to the medical regimen. For the next 72 hours, no status epilepticus or electrographic seizures were reported, although few occasional discharges were seen. Unfortunately, seizures recurred on day 14 and the patient succumbed to his multiple comorbidities on day 17. However, VNS was successful in acutely terminating status epilepticus for 72 hours in this critically ill patient when the standard therapies failed.

## 3. Discussion

Status epilepticus is a medical emergency with seizure activity lasting five minutes or longer [2]. This can be either one continuous seizure or multiple repetitive seizures with no recovery of consciousness in between [3]. Status epilepticus can include both generalized and partial seizures and has a high potential to cause brain injury so it therefore requires immediate drug therapy. The treatment protocol is intensive and includes benzodiazepines, anticonvulsants, and eventually anesthetics to medically induce coma when polypharmacy is exhausted [1].

The pathophysiology of NORSE remains unclear, with one study of 130 patients finding 52% of cases (n=67) to be cryptogenic in origin. In cases with an identifiable etiology, the most common was nonparaneoplastic autoimmune, specifically an anti-NMDA receptor antibody, followed by paraneoplastic causes [4]. Analysis of cerebrospinal fluid in NORSE patients also commonly demonstrated inflammatory pleocytosis, with infectious etiology being the third most common identified, although often without an identifiable organism [4]. Furthermore, similar to our patient, NORSE often occurs in the background of a preceding upper respiratory tract infection so one hypothesis is it is initiated in response to increased proinflammatory molecules in the brain following a viral infection [5–7].

NORSE is seen most frequently in young adults, though all ages can be affected. It does share similarities with other seizure syndromes which occur mainly in children, including Febrile Illness-Related Epilepsy Syndrome (FIRES) and Idiopathic Hemiconvulsion-Hemiplegia and Epilepsy Syndrome (IHHES), so some hypothesize that all may be extensions of a common disorder afflicting younger populations [4]. It is difficult to estimate the incidence of NORSE since it is often underreported as status epilepticus attributed to encephalitis. However, one Finnish study on SRSE estimated an annual incidence of 0.7/100,000 and a mortality rate of 36% [7]. So although cases of refractory status epilepticus are rare, they are associated with significant mortality. Prognosis for NORSE survivors is poor as 50% of survivors develop a chronic cognitive or functional disability and often have epilepsy moving forward [4].

Seizures in NORSE do not respond to the standard status epilepticus medications; hence the majority of patients are treated with an anesthesia-induced coma and monitored in ICUs [5]. One study estimates that nearly 40% of status epilepticus cases will be refractory to the first and second-line treatments of the aforementioned protocol [4]. The multiple toxicities of antiepileptic drugs have the potential to impart further damage to the already metabolically stressed brain. NORSE patients receive a median of five antiseizure medications during their treatment course yet 77% of cases culminate with administration of continuous anesthetics [4]. This trial-and-error approach may have deleterious effects; therefore, the high morbidity and mortality associated with NORSE may have an iatrogenic component rather than being entirely physiologic. Anesthetic use is particularly concerning since it is associated with poorer outcomes and increased mortality [4]. This relationship is not necessarily causal but still represents the need for alternative management strategies so there is great interest in aborting seizures with nonmedicinal devices.

One such option is vagus nerve stimulation (VNS), an FDA-approved therapy for refractory epilepsy that has been shown to decrease the frequency and severity of seizures [8, 9]. Unlike traditional antiepileptic drugs, VNS is hypothesized to control seizures by sending regular pulses to the brain through stimulation of the vagus nerve [8]. This different mechanism of action has a much lower side effect profile while still exerting a powerful antiseizure effect. One study reviewing seizure control in 65 patients with an implanted VNS found seizures decreased by 28% within 3 months of implantation [9]. This benefit extrapolates to long-term control with seizures decreasing by 36% within 6 months of implantation, 58% after 4 years, and 75% by 10 years [9].

Due to the efficacy of VNS in refractory epilepsy, there was interest in using it in refractory status epilepticus as an alternative nontoxic treatment option. We present the case of our patient with NORSE whose seizures were successfully controlled by VNS treatment.

This case report adds to current evidence on the effectiveness of using VNS for SRSE. Multiple case reports have described a benefit from implantation of VNS in the treatment of SRSE [10–15]. One such case report described a 24-year-old man with no history of epilepsy or neurological disorders presenting with delirium two days after onset of fever related to a mild upper respiratory tract infection [10]. On day 2 the patient developed left-sided clonic seizures which progressed to generalized tonic-clonic seizures. The frequency of seizures increased until patient entered status epilepticus. Status failed to be controlled by intravenous diazepam and phenytoin but eventually responded to intravenous propofol. The patient remained stable until day 14 when seizures recurred. Multiple therapies were tested, including thiopental, valproate, lamotrigine, steroid-pulse therapy, intravenous immunoglobulin, zonisamide, levetiracetam, topiramate, and midazolam but none provided lasting relief. Patient, presented 14 months later with refractory epilepsy and had deteriorated to the point of being bedridden, was incapable of verbal communication and required a feeding tube all while being treated with intravenous midazolam. Eventually a VNS was implanted in a final effort to control seizures. By day 10 the patient was successfully weaned off midazolam, and 2 months later he was seizure-free. The rapid modulation by VNS allowed reduction of intensive pharmacotherapy and contributed to seizure remission at one-year after implantation [10].

Another report described the case of a 30-year-old male with SRSE who could not remain on multiple antiepileptic medications including phenytoin, valproic acid, carbamazepine, and topiramate due to severe allergic reactions [13]. He was placed under a pentobarbital-induced coma without seizure control and after 9 days he underwent a VNS implantation. Within a day, EEG demonstrated resolution of seizures. During recovery the patient was able to be weaned off medications without triggering additional seizures and regained mental alertness and orientation with no residual compromise of his long-term memory [13].

Similarly, there was a case report of a 7-year-old girl with a history of thrombosis in the right internal cerebral vein and right thalamic bleeding 8 days after birth [11]. As a result of this early neurological insult, she developed epilepsy at 13 months of age. At 6 years she presented with refractory status epilepticus and spent 11 days in a medicine-induced coma. She subsequently underwent VNS placement with positive results. Her coma was withdrawn 3 days after implantation, her EEG normalized within 1 week, and at 13-month follow-up she was seizure-free and even her previous antiepileptic drugs were tapered. Although this is not a case of NORSE due to her history of epilepsy, this case shows the potential of VNS to acutely abort refractory status epilepticus, as well as impart a long-term benefit against seizures [11]. This is further demonstrated by the case report of a 23-year-old male who remained in SE for 3 weeks despite being on intravenous anesthetics. His SE terminated rapidly following VNS implantation and allowed anesthetics to be successfully weaned off and the patient fully recovered [9]. In another case report, a 13-year-old male had SRSE successfully aborted following VNS implantation [4]. Over the course of 1.5 years his seizures were better controlled in terms of both rate and severity [4].

One study reviewing current literature on all cases of VNS use in SRSE identified 17 studies in which a total of 28 patients were treated [15]. In patients with generalized SRSE, 76% displayed termination of SRSE with VNS use. Patients with focal SRSE demonstrated a less robust response, but 25% still responded to VNS insertion. Few adverse effects related to these VNS insertions were described. Despite a small sample size limiting the power of the study, this demonstrates strong evidence exists that there is improvement in seizure control with the use of VNS in generalized SRSE.

In most of these cases, like in our case, VNS was utilized late in the treatment plan, often following more than a week of status epilepticus and after multiple antiepileptic medications failed. However, the evidence shows that VNS has the capability to rapidly resolve status epilepticus within days of implantation. This discrepancy between the effectiveness of VNS and when it is to be employed must be studied with more cases.

We propose that VNS be considered earlier in the treatment course for SRSE as it is both efficacious and nontoxic. Despite our patient case being complicated by multiple confounding comorbidities, the case reports reviewed demonstrate a positive prognosis amongst patients treated with VNS which may offset the high mortality rates of SRSE syndromes. Furthermore, it would reduce the iatrogenic burden associated with status epilepticus polypharmacy treatments. In addition to having utility in halting the status, VNS has been shown to counteract the morbidity associated with SRSE. Patients receive the benefit of prophylaxis against future seizures and thereby require less long-term medications since epilepsy is common sequelae of SRSE disorders.

## 4. Conclusion

The medications used to control SRSE have several potential side effects which can further damage the brain so there is interest in finding an alternative treatment. VNS has been demonstrated by numerous case reports, including our case to be a safe and effective treatment for status epilepticus refractory to traditional treatment. Its lower side effect profile compared to antiepileptic medications reduces the toxicities vulnerable patients are exposed to, while offering a lasting protective effect against future seizures. Since there is typically extensive additional therapy complicating the complete assessment of VNS in status epilepticus, additional prospective study is warranted.

## Abbreviations

NORSE: New Onset Refractory Status Epilepticus
FDA: Food and Drug Administration
SE: Status epilepticus
SRSE: Super Refractory Status Epilepticus
EEG: Electroencephalography
MRI: Magnetic resonance imaging
VNS: Vagus nerve stimulation
FIRES: Febrile Illness-Related Epilepsy Syndrome
IHHES: Idiopathic Hemiconvulsion-Hemiplegia and Epilepsy Syndrome.
